# Progress in AAV-Mediated In Vivo Gene Therapy and Its Applications in Central Nervous System Diseases

**DOI:** 10.3390/ijms26052213

**Published:** 2025-02-28

**Authors:** Shuming Wang, Lin Xiao

**Affiliations:** 1 Institute for Brain Research and Rehabilitation, Guangdong Key Laboratory of Mental Health and Cognitive Science, Center for Studies of Psychological Application, South China Normal University, Guangzhou 510631, China; 2021010275@m.scnu.edu.cn; 2 Key Laboratory of Brain, Cognition and Education Sciences of Ministry of Education, South China Normal University, Guangzhou 510631, China

**Keywords:** gene therapy, adeno-associated virus, capsid engineering, aromatic L-amino acid decarboxylase deficiency, Parkinson’s disease, Alzheimer’s disease, Huntington’s disease, Canavan disease, central nervous system

## Abstract

As the blood–brain barrier (BBB) prevents molecules from accessing the central nervous system (CNS), the traditional systemic delivery of chemical drugs limits the development of neurological drugs. However, in recent years, innovative therapeutic strategies have tried to bypass the restriction of traditional drug delivery methods. In vivo gene therapy refers to emerging biopharma vectors that carry the specific genes and target and infect specific tissues; these infected cells and tissues then undergo fundamental changes at the genetic level and produce therapeutic proteins or substances, thus providing therapeutic benefits. Clinical and preclinical trials mainly utilize adeno-associated viruses (AAVs), lentiviruses (LVs), and other viruses as gene vectors for disease investigation. Although LVs have a higher gene-carrying capacity, the vector of choice for many neurological diseases is the AAV vector due to its safety and long-term transgene expression in neurons. Here, we review the basic biology of AAVs and summarize some key issues in recombinant AAV (rAAV) engineering in gene therapy research; then, we summarize recent clinical trials using rAAV treatment for neurological diseases and provide translational perspectives and future challenges on target selection.

## 1. Introduction

The existence of the BBB poses many challenges to drug discovery and development in neurological diseases. Numerous chemical medications are being developed to treat neurological disorders. Effective peripheral drug delivery requires these medications to cross the BBB and reach the brain at sufficient concentrations. Different neurological diseases have different mechanisms of pathological manifestation, which requires drugs to not only cross the BBB through systemic delivery but also to bind to the target site of the disease [1,2]. Gene therapy uses viral vectors that carry transgenes to effectively address two major challenges: the affinity for neural tissues and the permeability of the BBB. This is achieved, on the one hand, by optimizing the capsid genes of the viral vectors and, on the other hand, by incorporating functional genes that express therapeutic proteins into the vectors to treat diseases at the genetic level [3]. Gene therapy offers the advantage of providing a single-dose treatment and, thus, does not require repeated drug administration, leading to sustained therapeutic effects. This allows researchers to utilize targeted delivery systems specifically designed for the affected areas of the brain in various neurological diseases. By changing the route of administration, researchers can bypass the problem of BBB penetration in the treatment of neurological disorders without the need for capsid engineering. In summary, gene therapy is changing the strategy of neurological disease treatment.

Ideal gene delivery vehicles have the following characteristics: they are able to be effectively delivered to specific tissues without adverse effects on healthy tissues and organs; they have low immunogenicity and toxicity and do not integrate into the genome; they have long-lasting effects; and ideally, they achieve the treatment goal after one-time administration [4]. Although LVs occupy 10.7 kb of the RNA genome and are capable of loading large genes, they are known to have strong integration properties in the natural state and are commonly used in tumor cell therapy. Researchers are currently modifying the genome of LVs and making them suitable for in vivo clinical applications. For example, the integrase-deficient lentivirus (IDLV) is designed to prevent the integration of viral genes by knocking out the integrase gene that helps integrate the LV genome, but this can also reduce the gene expression; deleting the inhibitory factor histone deacetylase (HDAC) or incorporating the transcriptional enhancer Sp1 can improve the decreased gene expression levels resulting from the integrase knockout. Finally, LVs have the advantage of larger cargo capacity than rAAVs, so LVs with modifications may be a better gene vector vehicle in clinical trials in the future. Other virus types are less widely used because of their higher immunogenicity in the natural state, and safety concerns.

In in vivo gene therapies in clinical trials approved by the U.S. Food and Drug Administration (FDA), the use of rAAVs [5,6] is preferred over other viral vectors, such as LVs, adenoviruses (AdVs), and herpes simplex viruses (HSVs). AAV vectors are often used in clinical trials for in vivo gene therapy because of their advantageous biological properties. Certain serotypes of natural AAVs, such as AAV9 and AAV-rh10, can penetrate the BBB [7]. Many AAV serotypes are also capable of transducing neurons [8,9]. In addition, the genome of AAVs can be retained in non-dividing cells, including neurons, allowing for sustained transcription and expression. Most importantly, AAVs and rAAVs have a low risk of gene integration and oncology, although this property can be a disadvantage when transducing proliferative cells and losing their efficiency with cell cycles [10,11]. Despite advances in rAAVs, limitations for clinical requirements remain, including manufacturing, restricted cargo capacity, off-target effects, and efficiency problems. Innovations in rAAVs aim to address these challenges.

As an emerging therapeutic method, the first rAAV-based gene therapy for CNS disorders was registered in the European Union (EU) in 2022 for the treatment of aromatic L-amino acid decarboxylase deficiency (AADCD) [12], and the first rAAV-based gene therapy for spinal muscular atrophy (SMA), Zolgensma, was approved by the FDA in 2019. No LVs or other viral vectors of CNS in vivo gene therapy have been registered to date. Several therapies are in clinical trials and show promising clinical improvements, such as in Parkinson’s disease (PD). However, some therapies are still being studied in animal models or have not been investigated, particularly for some rare diseases. Overall, there is still much work to be done regarding the applications of gene therapy to CNS disorders.

In this review, we describe the biological properties of rAAVs, including the biological process of AAV transduction and the evolution from AAVs to rAAVs, and we provide the major directions of rAAV engineering aiming to solve cargo capacity, specificity, and efficiency problems in clinical applications. We also provide some overviews of ongoing and approved rAAV-based gene therapies and compare the differences between target genes in central nervous system diseases. We hope to clarify the promising targets for the development of rAAV gene therapeutics and provide guidance on the direction of research in order to help both basic scientists and clinical trial investigators explore the genetic contributions to complex diseases and rAAV engineering.

## 2. AAV Biology

### 2.1. The Structure of Natural AAVs

AAV belongs to the genus *Dependoparvovirus* of the *Parvoviridae* family. In the natural environment, AAV relies on a helper virus, such as AdV or HSV, to co-infect the same host cell to complete its replication cycle [13]. The wild-type AAV consists of a capsid composed of 60 subunits encasing a single-stranded DNA, thus forming a 20-faceted structure with an outer diameter of approximately 25 nm. In contrast to an LV, an AAV does not have an envelope outside its capsid. The AAV capsid is composed of three proteins, namely, viral capsid proteins 1, 2, and 3 (VP1, VP2, and VP3), which are organized in a ratio of 1:1:10 [14]. Importantly, the composition of these capsid proteins (i.e., the AAV serotype) determines the tissue transduction specificity, also known as AAV tropism [15,16]. Wild-type AAVs in humans and non-human primates (NHPs) have at least 12 different serotypes that have a preference for different tissues and cells [7,11,17,18].

**Figure 1:**
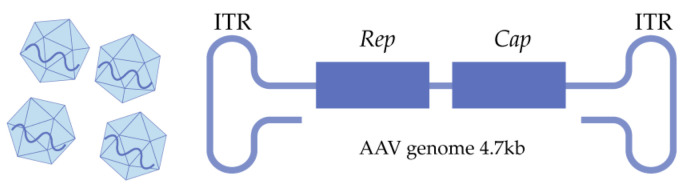
Natural AAV and ssDNA of 4.7 kb.

The genome of a wild-type AAV is a 4.7 kb single-stranded DNA (ssDNA) (Figure 1), and it consists of a pair of 145 bp at each end of the single strand, thereby forming a hairpin structure and several functional gene families: the rep gene encoding four isoforms of non-structural proteins, which are required for replication and packaging; the cap gene encoding VP1, 2, and 3 capsid proteins in one open reading frame (ORF); and the assembly-activating protein (AAP) in a separate ORF, which is necessary for capsid assembly [19].

### 2.2. AAV Transduction Journey

**Figure 2:**
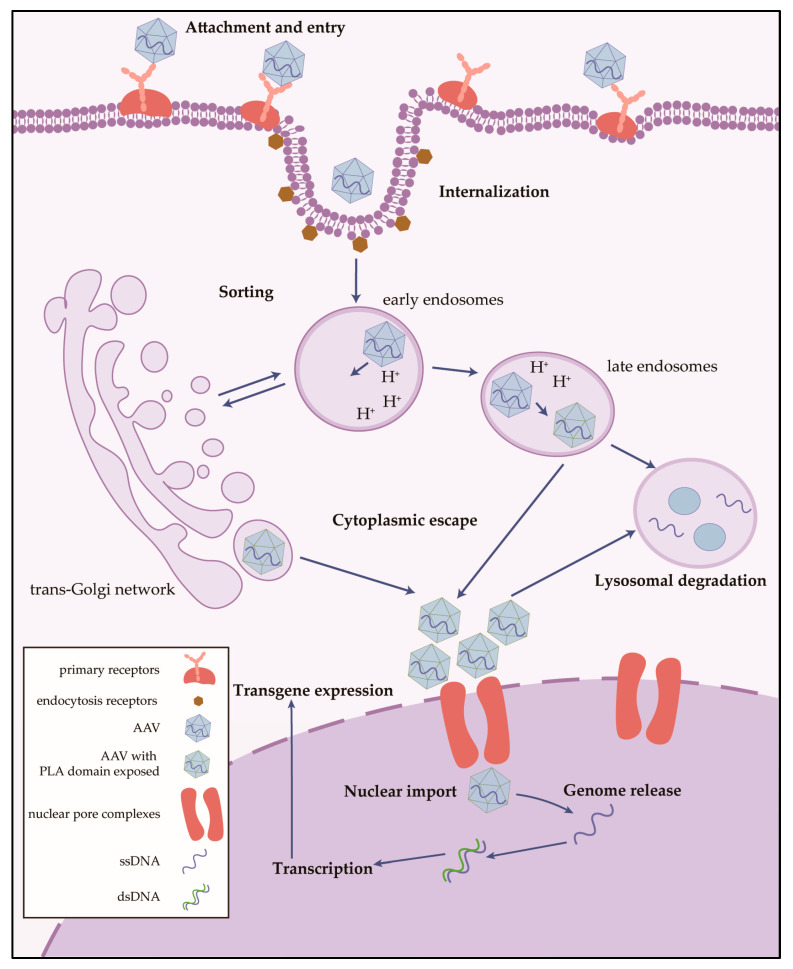
Cellular pathway of AAV transduction. The process is divided into multiple successive cellular events, including attachment, entry, internalization and sorting, cytoplasmic escape, nuclear import, genome release, and transgene expression. AAVs attach and enter host cells through receptor-mediated endocytosis and are sorted by the endocytosis vesicles including early endosomes (EEs) and late endosomes (LEs), and they shuttle to the trans-Golgi network. After escape, AAV particles accumulate around the perinuclear space and are capable of trafficking to the nucleus. Viral uncoating is required for the expression of the genome [20].

The mechanism by which AAVs retain tissue and cell preferences via capsids is not fully understood. To better explore infection and transduction, the process is divided into multiple successive cellular events, including attachment, entry, internalization and sorting, cytoplasmic escape, nuclear import, genome release, and transgene expression (Figure 2) [20]. AAVs attach and enter host cells through receptor-mediated endocytosis [21,22,23]. After entering the host cytoplasm, AAV particles are sorted by the endocytosis vesicles, including early endosomes (EEs) and late endosomes (LEs), and they shuttle to the trans-Golgi network (TGN) [24,25,26], followed by cytoplasmic escape. Then, AAV particles accumulate around the perinuclear space [27,28] and undergo uncoating and release the DNA genome after being transported into the nucleus through the nuclear pore complexes [28,29]. In fact, before AAVs uncoat and release their genomes, their structural properties determine the immunogenicity, tissue specificity, and transduction efficiency in the host [30]. A study based on human blood samples found that neutralizing antibodies (NAbs) against different AAV serotypes were present in healthy populations, with antibodies against AAV2 having the highest percentage [31], while another study showed that the NAb to AAV1 is the most common Nab, being in 74.9% of the population [32]. In the natural state of transduction, AAVs are nonpathogenic and do not cause any human disease.

Among the natural AAVs, AAV1, 2, 5, 6, 8, 9, rh8, and rh10 exhibit a strong affinity for the nervous system (Table 1). However, these AAVs do not specifically target neural tissues, and they show varying effectiveness across different types of cells within the nervous system [33,34]. Distinguishing and summarizing the accessibility, tissue specificity, neuronal cell specificity, and biomolecular mechanisms underlying the infection of the nervous system are of strategic importance for the modification of the capsid genome to achieve superior tissue specificity and neuronal cell specificity with high efficiency. More importantly, some natural AAVs, such as AAV9 and AAV-rh10, are able to cross the BBB and target the CNS via peripheral administration [7,9]. Understanding and studying the molecular mechanism of BBB permeability plays an important role in modifying and optimizing capsid proteins and promoting the development of rAAVs in gene therapy in CNS diseases.

During the attachment phase, the capsids of natural AAVs bind to primary receptors on the surface of the cell membrane and then interact with co-receptors; these primary receptors are mainly glycoproteins such as glycans, glycoconjugates, or sialic acid. For example, AAV2 interacts with heparan sulphate proteoglycans (HSPGs) [35,36]; AAV1, AAV5, and AAV6 primarily bind to N-linked sialic acid [37,38,39]; and rAAV9 interacts with N-linked galactose [40,41]. The various binding receptors seem to be the important factors influencing CNS tropism, as other AAVs lacking CNS tropism bind to different primary receptors. For example, AAV4 binds to O-linked sialic acid [37], and AAV3 can successfully transduce cells defective for heparin or heparan sulphate expression and is preferred for some haematopoietic cell types [42]. For other AAVs with CNS tropism, such as AAV8, rh8, and rh10, the primary receptors that they interact with have not yet been identified. Genome-wide screens have been applied to discover novel host proteins contributing to post-attachment. There are two key host proteins, namely, the type I transmembrane protein KIAA0319L and G protein-coupled receptor 108 (GPR108), and both facilitate the post-attachment step without affecting cell-surface binding [23,43,44]. Meanwhile, KIAA0319L has previously been reported as being the AAV receptor (AAVR) before [45]. Although KIAA0319L is an essential entry factor in many AAV serotypes, the mechanisms of its interaction with distinct AAV serotypes are different with regard to five polycystic kidney diseases (PKD1–5) domains on the ectodomain [17,46]. For example, AAV5 can be transduced via PKD1, AAV2 interacts with PKD2, and other serotypes such as AAV1 and AAV8 require a combination of PKD1 and PKD2 [17,46]. The heterogeneous binding sites with KIAA0319L strongly suggest that a variety of different AAV capsids should be considered during capsid engineering.

The internalization stage, followed by cell-surface binding, can occur via multiple receptor-mediated endocytosis or pathways including clathrin [47], caveolin [48], Rac family small GTPase 1 (RAC1) [22,49], or glycosylphosphatidylinositol-anchored protein-enriched endosomal compartments (GEECs) [50]. The involvement and effects of different endocytosis pathways vary among cell types [51,52], and how they function in neurons and glial cells is poorly understood. In addition, the studies have primarily focused on the AAV2 serotype, and more serotypes need to be studied in this regard. The endocytosed vesicles are then sorted and transported to EEs, LEs, and the TGN. This processing is partially serotype-specific; for example, the LE marker cation-independent mannose 6-phosphate (CI-MPR) was observed to colocalize with rAAV2 but not with rAAV8 [26], while the EE antigen1 (EEA1) colocalized with both of them [26,53]. This sorting step is also crucial for subsequent cytoplasmic escape, and the acidic pH environment of the endocytosis cargo facilitates a number of conformational changes in the capsid [54], especially the exposure of the hidden phospholipase A2 (PLA2) domain located in the N terminus of VP1 [55,56]. PLA2-mutant capsids fail to release from the TGN, which indicates that externalized PLA2 is necessary for cytoplasmic escape [57,58].

After the completion of cytoplasmic escape, AAV particles accumulate in the perinuclear region and translocate to the nucleus [27,59], during which the four basic regions (BR1-4) contained in the N terminus of VP1/2 carry nuclear localization signals, which are associated with the efficiency of AAV nuclear entry of AAV [60]. AAVs require nuclear entry signals to interact with the nuclear pore complexes (NPCs) [28], but different AAV serotypes bind to the NPCs through different binding constituents, such as importin-β and nucleoporins [29,61]. The AAV genome is then released from the capsid.

After cell entry, the released ssDNA is required to be converted into double-stranded DNA (dsDNA) via second-strand synthesis prior to transcription. The hairpin ends of the ITRs allow the virus to be recognized by the host DNA polymerase and to generate dsDNA; thus, modification and optimization of the ITR elements are carried out to facilitate the transcription and expression of the vector genome [62]. dsDNA can be maintained in the nucleus as episomal forms, leading to progressive degradation in dividing cells via cell division but prolonged transgene expression in non-dividing cells [63,64]. In in vitro experiments, there is a 0.1% probability that wild-type AAVs will integrate specifically into human chromosome 19 (19q13), the so-called AAVS1 site [65]. dsDNA from wild-type AAVs can be transcribed and replicated in the presence of a helper virus, AdV or HSV, or in a cellular stress environment; if it successfully assembles into viral particles and completes the replication cycle, the intact AAVs can be released from the host cells.

## 3. rAAV Engineering

### 3.1. rAAV Manufacturing

**Figure 3:**
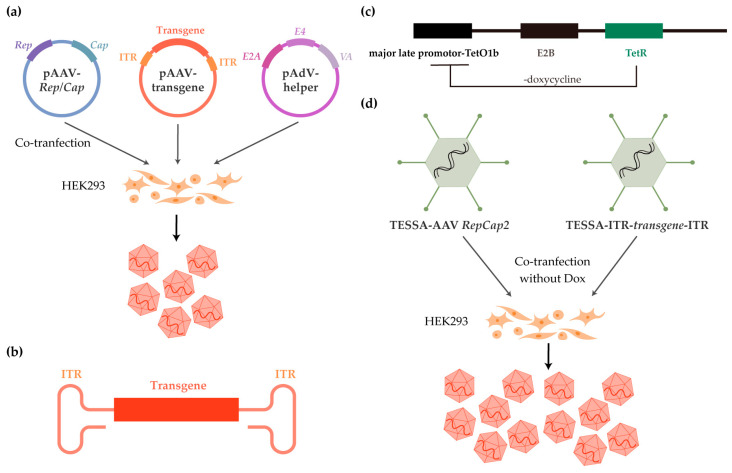
rAAV manufacturing. (**a**) Three-plasmid transient transfection of HEK293 cells; (**b**) Engineered rAAV genome with transgene of interest; (**c**) Schematic showing the mechanism of TESSA; (**d**) TESSA system for high scalability of rAAV manufacturing.

To meet the demand for the high-quality and bulk production of AAVs for research and clinical development, researchers have made some modifications to the natural AAV genome to produce rAAVs [66]. The most commonly used method is the transfection of plasmids containing all the required and desired genomes into HEK293T cells, which include the original viral genome and the helper genes in the helper virus (Figure 3) [67]. As AAV replication requires a helper virus to complete the replication and packaging of AAV particles, it is unable to achieve self-replication by reinfection [67]. Researchers have analyzed and identified several essential genes from the helper virus, including E1a, E1b, E2a, E4orf6, and viral-associated RNA (VA) genes. As 293T cells can synthesize E1a and E1b independently [68], the final constructed helper plasmid includes all important helper genes, except for E1a and E1b (Figure 3a). In addition, the direct transfection of helper plasmids instead of co-infection with a helper virus avoids the contamination of other viral particles in the rAAV production process. In the AAV genome, the ITRs are essential for generating dsDNA and are therefore necessary for maintaining long-term expression of the transgene in gene therapy. In contrast, the rep and cap genes are only required for producing the enzymes necessary for replication, as well as for generating the VPs needed for capsid packaging during the rAAV production process [69]. Since these two components serve different roles in rAAV production or clinical treatment, the original AAV genome is split and constructed into two separate plasmids, one termed the packaging plasmid, which carries the rep and cap genes, and the other called the vector plasmid, which carries the gene of interest flanked by ITRs.

In this three-plasmid system, the vector plasmid, packaging plasmid, and helper plasmid are co-transfected into HEK293T cells for the large-scale production of rAAVs, and this has been modified to a two-plasmid system by combining the helper plasmid and the packaging plasmid into a single plasmid, pDG [70,71]. Based on the double transfection system, the pQT packaging system was developed to improve good manufacturing practice (GMP) compatibility and flexibility for clinical manufacturing [72]. The rAAVs packaged by the triple or double system consist of the capsids (assembled and transcribed according to the packaging plasmid), the ITR sequences from the original viral genome, and the ORFs replaced by transgenes. The entire rAAV manufacturing strategy is based on the biology of the AAV transduction pathway and practical requirements. When rAAVs are used for gene therapy, only the inserted transgenes are retained in the target tissues (Figure 3b). These transgenes produce proteins that either compensate for defective protein expression, inhibit mutant proteins, or provide other therapeutic benefits, depending on the specific clinical and research objectives.

This transient transfection approach is flexible due to the easy and efficient generation of rAAVs with diverse transgenes and capsids by simple plasmid construction, but scalability is limited to 200 L even when using suspension host cells. It also suffers from high cost. Viral infection-based approaches, including the baculovirus expression vector system (BEVS) [73,74,75], stable cell lines combined with an AdV-AAV hybrid [76,77,78] or recombinant HSV (rHSV) system [79,80] have been optimized for large yields. However, baculovirus is genetically unstable [81,82] and inherently unable to assemble AAV capsids in a proper 1:1:10 ratio [83,84]; creating stable cell lines with specific vector genomes is time-consuming and thus, inflexible; the involvement of an AdV-AAV hybrid or rHSV also requires robust downstream purification to eliminate unrelated viral contaminants [78].

Developing safe and effective rAAV production methods should address issues of flexibility, scalability, and quality. A recent breakthrough, the tetracycline-enabled self-silencing adenovirus (TESSA) system, perfectly addresses these problems [85]. Based on the viral infection approach, a helper AdV was designed to introduce an inducible tetracycline repressor (TetR) binding site, tetracycline operator (TetO), into its major late promoter (MLP) and TetR under its transcriptional control (Figure 3c). In the absence of doxycycline (Dox), MLP-TetO is inhibited by TetR, the modified AdV is limited to genome amplification, and early gene expression functions as a facilitator of AAV packaging (Figure 3d). This system is suitable for large-scale rAAV production without AdV contamination.

### 3.2. Capsid Innovation

As capsids determine the transduction and efficiency of rAAV and the expression of the transgene of interest. Capsid engineering has emerged as an efficient strategy for developing rAAVs for clinical applications. While natural AAVs have advantageous properties for transducing human tissues, it is important to note that 40–80% of people have antibodies against wild-type AAV serotypes [30,31]. As a result, rAAVs derived from natural AAVs remain the primary choice for optimizing AAV vectors. However, AAV serotypes in non-human species have limitations in their ability to transduce human tissues [86,87].

There are three major approaches to capsid engineering: rational design, directed evolution, and machine learning-based capsid innovation. The rational design approach involves making structural modifications at specific sites on AAV capsids. In contrast, directed revolution applies selective pressure to various capsid variants to achieve clinical applications such as an increased rAAV yield, enhanced transduction, immune response evasion, or specific cell/tissue targeting. Recently, deep learning techniques combined with bioinformatic prediction have provided a novel avenue for advancing capsid engineering [88].

**Figure 4:**
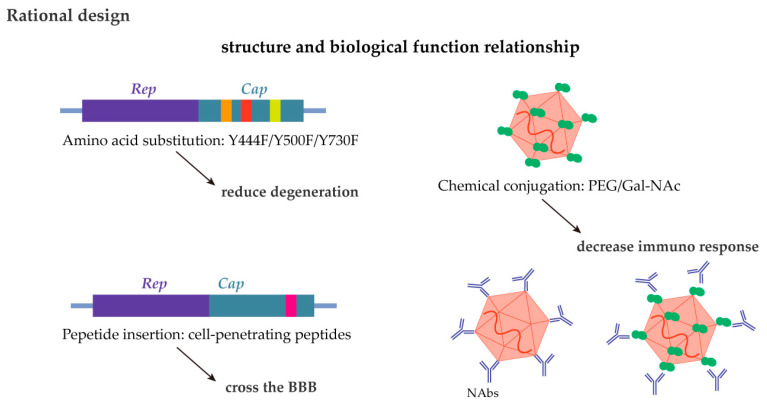
Rational capsid design utilizes knowledge of the structure–function relationship to achieve BBB penetration, efficiency, and immune evasion.

Although rational design can be limited by our basic knowledge of the relationship between the rAAV structure and biological function, numerous advances via rational design have improved rAAV delivery efficiency and specificity (Figure 4). For example, studies on exploring specific amino acid residues found that the phosphorylation of tyrosine residues on rAAV2 capsids contributed to the degradation of nuclear import [89,90]. Modifying these residues to phenylalanine (Y444F/Y500F/Y730F) increased transduction in the CNS [91]. Studies have also found that cell-penetrating peptides facilitate rAAV9 in crossing the BBB [92]. By inserting these peptides into rAAV9, CNS transduction was enhanced following intravenous (i.v.) delivery. To decrease the host immune response, conjugating the rAAV capsid with biotin-polyethylene glycol (PEG) and N-acetylgalactosamine (GalNAc) may help evade Nabs, leading to longer and more efficient transgene expression [93,94,95].

**Figure 5:**
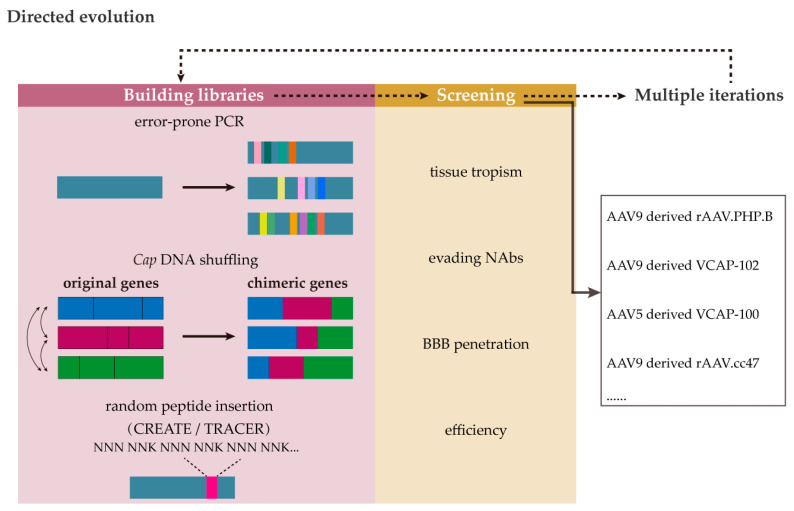
Directed evolution for capsid engineering. Capsid library construction methods include error-prone PCR, DNA shuffling, and random peptide insertion. After screening capsids with preferred properties, the capsid genome is used to build a smaller library for the next round of screening.

The directed evolution strategy involves first building a library of randomly mutated AAV variants, followed by isolating specific mutants with prospected properties such as BBB penetration, tissue tropism, or enhanced transduction (Figure 5) [11,96]. Methods for generating diverse libraries consist of error-prone PCR and a staggered extension process [97], a DNA shuffling-based approach [98,99], the random peptide insertion into surface regions [100,101], etc. For example, the Cre recombination-based AAV targeted evolution (CREATE) system, which attaches short-length peptides to the rAAV9 capsid, was used to generate an AAV-PHP.B. variant that could penetrate the BBB in C57BL/6 mice but not in other mice strains or NHP [100,102,103]. Further studies need to consider cross-species transfection problems. A novel rAAV evolution platform termed TRACER (tropism redirection of AAV by cell-type-specific expression of RNA) was then created through random peptide insertions in a cell-type-specific manner [101]. A study reported that 10 variants based on rAAV9 showed over 400-fold higher brain transduction following systemic administration [104].

Machine learning (ML) has been widely used in biomedical research, such as image analysis and drug discovery [105,106]. Recent studies have utilized ML to analyze the rAAV2 capsid; they deciphered the landscape and single-codon substitutions, and they used ML to design precise multi-mutated variants based on their tropism function [88]. However, these results are still at the theoretical stage and need to be demonstrated experimentally for clinical applications [107]. The application of ML is becoming a promising engineering strategy, and it is less time-consuming and laborious than traditional experimental methods [108].

### 3.3. rAAV Genome Innovation

The rAAV genome contains ITRs, a transgene of interest, and regulatory components. These components cumulatively affect cell-type specificity and the durability and efficiency of the transgene expression. The use of combined strategies to engineer the rAAV genome and capsid can effectively overcome these problems in clinical settings.

The natural AAV genome consists of ssDNA, which needs to synthesize double-strand DNA before gene transcription. The transduction rate is limited by this second-strand step. Some studies have modified the ITRs by mutating one of them, converting it into self-complementary DNA [109,110]. However, this strategy reduces the genome packaging capacity by half and increases the risk of immune responses due to elevated levels of transgene products [111,112].

Although ubiquitous promoters such as the cytomegalovirus early enhancer/cytomegalovirus promoter (CMV) and the cytomegalovirus early enhancer/chicken β-actin promoter (CBA) are commonly and universally used to trigger enhanced expression [113,114], adding a specific promoter allows for transgene expression in targeted cells and less expression in other infected tissues without the initiation of downstream genes. For example, a study reported that a rAAV9 vector with survival motor neuron 1 (SMN1) promoter can drive specific SNM1 expression in neurons [115]. Another study revealed that a 229 bp of the mouse methyl-CpG-binding protein 2 (MeCP2) promoter provided durable expression specifically in neurons [114]. However, tissue-specific promoters may drive less active expression in diseased target cells and have fewer outcomes. Many full-length tissue-specific promoters such as the glial fibrillary acidic protein (2.2 kb) are too large to insert in rAAV vectors, as opposed to ~300 bp of CMV and 800 bp of CBA [116]. Overall, the promoter in the transgene genome provides another promising approach to achieving cell-type-specific transgene expression. Optimizing the engineering of capsid tropism and the promoter can collectively achieve special transduction in desired cells and reduce off-target effects. In addition, gene therapy clinical trials have also employed various enhancers to increase transgene expression [117]. A recent study reported a machine learning-guided platform to engineer synthetic cis-regulatory elements (CREs), with 200 bp capable of driving gene expression in specific programmed cell types [118]. One of the synthetic CREs (Syn1) was neuron-specific and was validated in vivo, with highly unique and effective expression in neurons but not in off-target cells including astrocytes and microglia. This work, combined with recent works for designing synthetic enhancers, demonstrates that machine learning can serve as a valuable catalyst for improving the specificity and efficiency of gene therapies [118,119,120,121].

**Figure 6:**
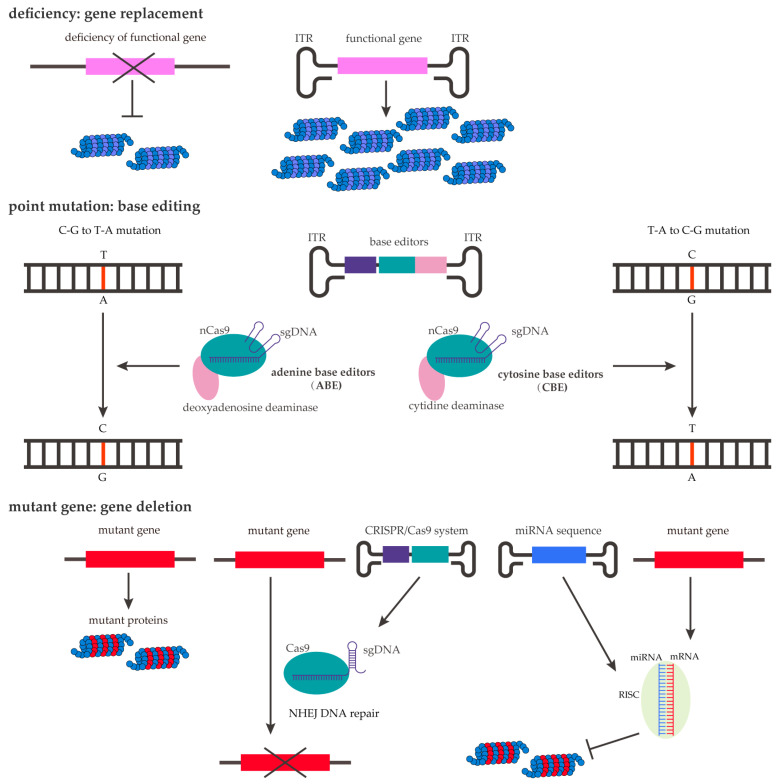
Etiology-specific transgene design strategies such as gene replacement, base editing, and lowering gene expression.

The transgene inserted into the vector is primarily determined by the genetic cause of the diseases (Figure 6). Monogenic diseases are the most suitable diseases for gene therapy. Many neurological diseases have a gene mutation or deletion or expression of pathologic proteins [122,123]. Typically, gene expression deficiency can be restored via the over-expression of rAAV vectors, i.e., gene replacement therapy. For diseases with a specific genetic mutation, base editors combined with rAAV delivery are used. For diseases caused by the pathological mutant protein, the rAAV transgene aims to delete mutant genes or interfere with their transcription [124,125,126]. The rAAV genome has a load of less than 4.7 kb; thus, determining how to insert genes, as well as regulatory elements, in this limited load is of great importance. Several approaches have attempted to overcome this load capacity, such as by splitting one large construct into two different rAAV vectors and reassembling them into a full-length functional protein via a split intein [127]. The all-in-one platform is another strategy used to achieve this goal [128,129,130]; studies have developed a compact bacterial clustered regularly interspaced palindromic repeat (CRISPR)/dCas9-based repressor system that has been shown to robustly silence the expression of apolipoprotein E4 (APOE4), a risk factor highly expressed in late-onset Alzheimer’s disease [127,131].

## 4. Gene Therapy and Clinical Applications in Central Nervous System Diseases

Based on the understanding of AAVs and rAAVs and the developments in their engineering, plenty of clinical trials of gene therapy based on rAAV vectors have been approved, and some have been introduced to the market. In this section, we summarize recent clinical applications of rAAVs in several central nervous system diseases and provide our translational views on target selection (Table 2).

### 4.1. Aromatic L-Amino Acid Decarboxylase Deficiency (AADCD)

AADCD is an autosomal recessive disorder caused by mutations in the dopa decarboxylase gene, resulting in a deficiency of the AADC enzyme [122,132]. It functions in dopamine and serotonin production followed by norepinephrine and epinephrine synthesis [132]. All of these are essential neurotransmitters in the CNS, and reduced levels of these components cause severe symptoms including muscular hypotonia, dystonia, oculogyric crisis, developmental delay, and autonomic dysfunction [133]. Symptoms occur 2.7 months after birth, and the average time of diagnosis is 3.5 years [132]. Patients always miss all developmental milestones and die before 10 years of age [122,133]. In 2012, rAAV2-human *AADC* (*hAADC*) gene therapy was first applied in children with AADCD via stereotactic infusion. Patients showed improved motor and mental performance [134], and a follow-up phase 1/2 trial also found enhanced clinical motor function and improved AADC activity in older patients than before [135]. Improvements in patient symptoms were found to last for over 5 years, and sustained dopamine improvement was observed via positron emission tomography (PET) and neurotransmitter analyses; this encouraged the approval of eladocagene exuparvovec for AADCD patients aged over 18 months in the EU [12].

### 4.2. Parkinson’s Disease (PD)

Parkinson’s disease is the second most common neurodegenerative disease, affecting more than 6 million people [136,137]. It is characterized by a lower level of dopa-mine caused by the progressive loss of dopamine neurons in the striatum region [138]. Motor and nonmotor symptoms, such as cognitive impairment and emotional issues can be observed in moderate and severe PD progress [136]. As PD has the same neurotransmitter deficiency as AADCD, this has encouraged the development of the same complementary strategy for both diseases. Phase 1 clinical studies of AAV2-*hAADC* administered via a bilateral injection into the putamen demonstrated its safety and tolerability; patients displayed increased dopamine production and sustained improvement in motor functions [139,140]. These efficient results encouraged a multicenter-controlled randomized placebo sham phase 2 trial (RESTORE-1, NCT03562494).

### 4.3. Canavan Disease (CD)

CD is an autosomal recessive inherited neurodegenerative disease caused by an aspartoacylase (*ASPA*) mutation. ASPA enzyme catalyzes N-acetylaspartate (NAA) into acetate and aspartate in oligodendrocytes [141]. The loss of function in the enzyme contributes to spongiform decreasing and NAA accumulating, followed by an absence of myelin in the CNS [142]. Patients find it hard to learn to talk and walk, show delayed motor milestones, and typically die before 10 years of age [143]. In 2012, researchers treated patients with an AAV2 vector containing the human *ASPA* (*hASPA*) gene via multi-region intraparenchymal administration. However, efficiency was not as expected, and NAA change was modest and not uniform throughout the brain [144]. By optimizing vector design and delivery methods, treatment based on intravenous (i.v.) administration alleviated the major disease symptoms in the mice model [145]. Currently, two phase 1/2 trials are ongoing (NCT04833907 and NCT04998396); these two trials differ in terms of vectors (rAAV-Olig1 and rAAV9) and delivery methods (intraventricular and i.v.).

### 4.4. Alzheimer’s Disease (AD)

AD is the most common progressive degenerative neurological disease, and up to 13 million people are expected to be affected by 2050 [146]. Aging is an important factor in the progression of AD. Although AD is not a conventional genetic disease, numerous studies have shown that apolipoprotein E (*APOE*) is a major risk gene for the progression of AD, with *APOE4* increasing the risk of AD [147] and *APOE2* preventing AD [148]. Based on the protective role of *APOE2*, with strong experimental evidence in AD [149,150] and by optimizing delivery routes in NHPs [151], a phase 1/2 clinical study (NCT03634007) is being conducted in the USA. The study has recruited 15 patients, with AAVrh10-*APOE2* treatment administered into the cerebrospinal fluid (CSF), and it will be completed in 2024. In a multicenter phase 2 trial (NCT00876863) [152], AAV2-nerve growth factor (*NGF*) was intracerebrally injected into the nucleus basalis. AAV2-*NGF* was found to be safe and well tolerated over 24 months but showed no improvements in clinical outcomes or selected AD biomarkers compared to the placebo group.

### 4.5. Huntington’s Disease (HD)

HD is caused by CAG expansion in exon 1 of the Huntingtin (*HTT*) gene. In healthy people, the number of CAG repeats is less than 27, while HD patients have more than 35 CAG repeats. Individuals carrying 27 to 40 CAG repeats do not develop the disease; instead, their offspring may inherit it [153]. The extension of CAG translates into polyglutamine at the N-terminal region of the HTT protein, leading to misfolded proteins with a gain of function [123]. The mutated HTT protein mainly attacks the medium spiny neurons in the striatum, as well as leading to astrogliosis and cell loss in the cortex with the process of the disease progression [154,155]. HD occurs in middle-aged people, with hallmarks of movement symptoms such as involuntary movements, variable degrees of rigidity, and incoordination. The main treatment strategy in gene therapy aims to lower the mutated HTT protein (mHTT) levels [123], and there are two ongoing phase 1/2 clinical studies being carried out by UniQure Biopharma, Amsterdam, The Netherlands (NCT04120493 in the U.S. and NCT05243017 in the EU). Both trials applied rAAV5-micro RNAs targeting *HTT (miHTT)* (AMT-130) through multi-site injections in the striatum, and NCT04120493, which started earlier, showed a decline in CSF mHTT at 12 months post-treatment [156].

### 4.6. Spinal Muscular Atrophy (SMA)

SMA is an autosomal recessive inherited neurodegenerative disease caused by low levels of the spinal motor neuron (SMN) protein, which is essential for the viability of motor neurons [157]. There are four forms of SMA, and SMA type 1 (SMA1) is the most severe form [158]. Symptoms of SMA1 disease appear 6 months after birth, and patients consistently miss all motor milestones and die before the age of 2 [159,160]. SMN protein is primarily encoded by *SMN1* and partially by *SMN2*; *SMN2* produces a smaller SMN protein, and the copy number of *SMN2* is negatively correlated with the severity of SMA [161]. Infants with *SMN1* deletions and two copies of *SMN2* have a 97% risk of SMA1. In 2014, the first in vivo gene therapy was initiated with a single dose of self-complementary rAAV9-CBA promoter-*hSMN1* via intravenous infusion. In 2017, it was reported that treated patients had a reduced need for respiratory support and improved motor function [162]. Two phase 3 clinical trials have evaluated the safety and efficacy of this treatment, [163,164] and the rAAV9-*hSMN1* therapy (Zolgensma) was already approved in 2019. A phase 3 clinical trial in 2022 has reported that infants at risk for SMA1 who received treatment prior to symptom onset achieved developmental milestones compared to untreated infants [165].

### 4.7. Sphingolipid Metabolic Disorders

Sphingolipid metabolic disorders are a subgroup of lysosomal storage disorders with toxic sphingolipid accumulation in various organs. Here we review some forms, including Tay–Sachs disease, Sandhoff disease, metachromatic leukodystrophy, and Krabbe disease, all of which have the CNS as the most affected organ [166].

Tay–Sachs disease and Sandhoff disease are also GM2-gangliosidoses resulting from loss-of-function mutations in the ubiquitously expressed gene encoding β-N-acetylhexosaminidase (HEX). HEX is a dimer consisting of subunits α and β (HEXA), β and β (HEXB), or α and α (HEXS). Tay–Sachs disease is caused by mutations of *HEXA*, and Sandhoff disease is caused by mutations of *HEXB* [167]. Co-delivery of *HEXA* and *HEXB* is commonly used in treatment. In one phase 1 clinical trial, rAAV8.rh8-*HEXA* and -*HEXB* were infused intrathecally, and the combined vectors were found to be well tolerated [168]. Currently. Two phase 1/2 trials are currently ongoing (NCT04798235 and NCT04669535), with NCT04798235 using a bicistronic vector rAAV9-*HEXB-P2A-HEXA* and NCT04669535 with rAAV8.rh8-*HEXA* and *-HEXB* vectors.

Metachromatic leukodystrophy is an autosomal recessive disorder caused by mutations in the arylsulfatase A (*ARSA*) gene. Loss of function of the enzyme contributes to the accumulation of sulfatides in the CNS and subsequent myelin damage [169]. One ongoing phase 1/2 clinical study is being carried out to intracerebral infuse rAAVrh10-*ARSA* controlled by a CMV/CBA hybrid promoter.

Krabbe disease is another demyelinating disease resulting from accumulation of galactosylceramides, which is caused by the decreased activity of galactosylceramidase (GALC) [170,171]. Similar to other gene replacement therapies, a clinical trial (NCT04771416) applied rAAVhu68 to carry *GALC* gene via intra cisterna magna after safety and efficacy evaluation of viral delivery [172].

### 4.8. Microcephaly–Capillary Malformation Syndrome (MIC-CAP)

MIC-CAP is an autosomal recessive inherited disease caused by biallelic mutations of the signal transducing adaptor molecular-binding protein (*STAMBP*) gene, resulting in a deficiency of STAMBP [173]. STAMBP is an endosomal deubiquitinating enzyme, and its loss causes microcephaly and generalized cutaneous capillary malformations at birth, early-onset intractable epilepsy, and developmental delay in patients [174,175]. MIC-CAP was first found in 2011, and 24 patients have been reported [176,177,178]. No clinical trials of gene therapy are undergoing so far. A preclinical study established a mouse model by CNS-specific conditional knockout *STAMBP* gene [179]; mice showed neurological defects and growth retardation. Intracerebroventricular (ICV) injection of AAV9-EF1a-*STAMBP* rescued the neurological symptoms and prolonged the survival of the mice. Further large animal models are needed to validate the safety and proper dosage of the treatment.

**Table 2:** Clinical trials of rAAV-based gene therapies for neurological diseases.

Disease	AAV Serotype	Gene	Delivery and Dose	Year	Clinical Phase
AADCD	rAAV2	*hAADC*	bilateral intraputaminal 1.6 × 10^11^ vg in total	2012	Phase 1/2 [134]
	rAAV2	*hAADC*	bilateral intraputaminal injection 1.81 × 10^11^ vg in total	2017	Phase 1/2 [135] NCT01395641
PD	AAV2	*hAADC*	bilateral injection into the putamen up to 3.6 × 10^12^ vg	2010	Phase 1 [140]
	AAV2	*hAADC*		2018–present	Phase 1 NCT03562494
	AAV2	*GDNF*	interstitial administration	2013	Phase 2
	AAV2	*hGDNF*	interstitial administration	2020–present	Phase 1b NCT04167540
CD	AAV2	*ASPA*	multi-region intraparenchymal administration 9 × 10^11^ vg in total	2012	[144]
	rAAV-Olig1	*ASPA*	intraventricular 3.7 × 10^13^ vg in total	2021–present	Phase 1/2 NCT04833907
	rAAV9	*ASPA*	intravenous administration dose-finding phase	2021–present	Phase 1/2 NCT04998396
AD	AAV2	*NGF*	intracerebrally injection into the nucleus basalis	2018	phase 2 [152] NCT00876863
	AAVrh10	*hAPOE2*	CSF infusion	2019–present	Phase 1/2 NCT03634007
	AAV2	*BDNF*	stereotactic injection into the brain	2022–present	Phase 1 NCT05040217
HD	rAAV5	*miHTT*	multi-site injection in the striatum 6 × 10^12^ vg or 6 × 10^13^ vg	2019–present	Phase 1/2 NCT04120493
	rAAV5	*miHTT*	multi-site injection in the striatum 6 × 10^12^ vg or 6 × 10^13^ vg	2019–present	Phase 1/2 NCT05243017
SMA1	rAAV9	*hSMN1*	intravenous administration low dose: 6.7 × 10^13^ vg per kilogram high dose: 2.0 × 10^14^ vg per kilogram	2014–2017	Phase 1 [162] NCT02122952
	rAAV9	*hSMN1*	1.1 × 10^14^ vg per kilogram	2017–2019	Phase 3 [163] NCT03306277
	rAAV9	*hSMN1*	1.1 × 10^14^ vg per kilogram	2018–2020	Phase 3 [164] NCT03461289
	rAAV9	*hSMN1*	1.1 × 10^14^ vg per kilogram	2018–2021	Phase 3 [165] NCT03505099
GM2- gangliosidoses	rAAV9	*HEXA-P2A-HEXB*	intrathecal	2021–present	Phase 1/2 NCT04798235
	rAAVrh8	*HEXA, HEXB*	bilateral thalamic, dual intrathecal 1:1 ratio of AAVrh8-*HEXA* and -*HEXB*	2021–present	Phase 1 NCT04669535
Metachromatic leukodystrophy	AAVrh10	*ARSA*	intracerebral low dose: 1.0 × 10^12^ vg high dose: 4.0 × 10^12^ vg	2014–present	Phase 1/2 NCT01801709
Krabbe disease	rAAVhu68	*hGALC*	intracisternal magna low dose: 1.5 × 10^11^ vg high dose: 5.0 × 10^11^ vg	2022–present	Phase 1/2 NCT04771416

## 5. Conclusions and Future Directions

Unlike chemical drugs, which have pharmacological mechanisms, and antibody preparations such as vaccines, gene therapy affects the pathophysiology process at the genetic level, so it can only work when viral vectors carrying therapeutic genes transfect and transduce lesioned areas. This new approach also differs from those others because it does not require the testing of the pharmacokinetics (PK) or pharmacodynamics (PD) of chemical drugs, and the gene-drug does not undergo the absorption, distribution, metabolism, and elimination processes. Moreover, the toxicity of viruses depends on the immune response to the viral capsid. Gene therapy clinical trials also require evidence of the mechanism of concept via in vivo equipment such as PET scans.

Gene therapy is widely used in the treatment of monogenetic diseases, which means that there is a clear causal relationship between the gene mutation and disease onset. Thus, more studies on the causes of disease need to focus on the most susceptible genes. To construct a good animal or cell model, the model should not only mimic part of the pathological manifestations of the disease but should also verify the related gene mutation through the extensive screening of risk genes in clinical samples. In addition, the onset of many diseases is caused by a combination of susceptibility genes and the environment, and, unlike monogenetic diseases, some diseases with a complex etiology are not amenable to gene therapy. Clinical trials for such diseases use in vivo gene therapy strategies to compensate for the expression of some proteins that have been shown to alleviate the pathological conditions, but the treatment outcomes are not satisfactory, even though AAV vectors mediate sustained and obvious changes in these functional proteins. This also encourages us to focus on the risk genes for diseases with complex etiology to better mimic the development of such diseases.

## Figures and Tables

**Table 1:** Comparison of biomolecular interactions between natural AAVs with and without CNS tropism.

CNS Tropism	AAV Serotype	Primary Receptors	PKD Selection of AAVR
Yes	AAV1	N-linked sialic acid	PKD1 and PKD2
	AAV2	HSPGs	PKD2
	AAV5	N-linked sialic acid	PKD1
	AAV6	N-linked sialic acid	Unknown
	AAV8	Unknown	PKD1 and PKD2
	AAV9	N-linked galactose	Unknown
	AAVrh8	Unknown	Unknown
	AAVrh10	Unknown	Unknown
No	AAV3	HSPGs-independent	Unknown
	AAV4	O-linked sialic acid	Unknown
	AAV7	Unknown	Unknown

## Data Availability

Not applicable.

## Abbreviations

BBB	blood–brain barrier
CNS	central nervous system
AAV	adeno-associated virus
LV	lentivirus
FDA	Food and Drug Administration
AdV	adenovirus
HSV	herpes simplex virus
IDLV	integrase-deficient lentivirus
HDAC	histone deacetylase
VP1, 2, 3	viral capsid protein 1, 2, 3
NHPs	non-human primates
ssDNA	single-stranded DNA
ORF	open reading frame
AAP	assembly activating protein
EEs	early endosomes
LEs	late endosomes
TGN	trans-Golgi network
NAbs	neutralizing antibodies
HSPGs	heparan sulphate proteoglycans
GPR108	G protein-coupled receptor 108
AAVR	AAV receptor
PKD	polycystic kidney diseases
RAC1	Rac family small GTPase 1
GEECs	glycosylphosphatidylinositol-anchored protein-enriched endosomal compartments
CI-MPR	cation-independent mannose 6-phosphate
EEA1	EE antigen1
PLA2	phospholipase A2
BR	basic regions
NPCs	nuclear pore complexes
dsDNA	double-stranded DNA
VA	viral-associated RNA
BEVS	baculovirus expression vector system
rHSV	recombinant HSV
TESSA	tetracycline-enabled self-silencing adenovirus
TetR	tetracycline repressor
MLP	major late promoter
Dox	doxycycline
GMP	good manufacturing practices
i.v.	intravenous
PEG	polyethylene glycol
GalNAc	N-acetylgalactosamine
CREATE	Cre recombination-based AAV targeted evolution
TRACER	tropism redirection of AAV by cell-type-specific expression of RNA
ML	machine learning
CMV	cytomegalovirus
CBA	chicken β-actin promoter
SMN1	survival motor neuron 1
MeCP2	methyl-CpG-binding protein 2
CREs	cis-regulatory elements
CRISPR	clustered regularly interspaced palindromic repeats
ABE	adenine base editors
CBE	cytosine base editors
NHEJ	non-homologous end-joining
RISC	RNA-induced silencing complex
APOE	apolipoprotein E
AADCD	aromatic L-amino acid decarboxylase deficiency
*hAADC*	human *AADC*
PD	Parkinson’s disease
GDNF	glial derived neurotrophic factor
CDNF	cerebral dopamine neurotrophic factor
CD	Canavan disease
ASPA	aspartoacylase
*hASPA*	human *ASPA*
NAA	N-acetylaspartate
AD	Alzheimer’s disease
CSF	cerebrospinal fluid
NGF	nerve growth factor
HD	Huntington’s disease
HTT	Huntingtin protein
mHTT	mutated HTT protein
*miHTT*	micro RNAs targeting *HTT*
SMA	spinal muscular atrophy
SMN	spinal motor neuron
SMA1	SMA type 1
HEX	β-N-acetylhexosaminidase
ARSA	arylsulfatase A
MIC-CAP	microcephaly–capillary malformation syndrome
ICV	intracerebroventricular
PK	pharmacokinetics
PD	pharmacodynamics
PET	positron emission tomography
